# Prognostic Value of Radiomic Analysis Using Pre- and Post-Treatment ^18^F-FDG-PET/CT in Patients with Laryngeal Cancer and Hypopharyngeal Cancer

**DOI:** 10.3390/jpm14010071

**Published:** 2024-01-05

**Authors:** Joon Ho Choi, Joon Young Choi, Sang-Keun Woo, Ji Eun Moon, Chae Hong Lim, Soo Bin Park, Seongho Seo, Yong Chan Ahn, Myung-Ju Ahn, Seung Hwan Moon, Jung Mi Park

**Affiliations:** 1 Department of Nuclear Medicine, Soonchunhyang University Bucheon Hospital, Bucheon 14584, Republic of Korea; 2 Department of Nuclear Medicine, Samsung Medical Center, Sungkyunkwan University School of Medicine, Seoul 06351, Republic of Korea; 3 Division of Applied RI, Korea Institutes of Radiological and Medical Sciences, Seoul 01812, Republic of Korea; 4 Department of Biostatistics, Soonchunhyang University Bucheon Hospital, Bucheon 14584, Republic of Korea; 5 Department of Nuclear Medicine, Soonchunhyang University Seoul Hospital, Seoul 04401, Republic of Korea; 6 Department of Electronic Engineering, Pai Chai University, Daejeon 35345, Republic of Korea; 7 Department of Radiation Oncology, Samsung Medical Center, Sungkyunkwan University School of Medicine, Seoul 06351, Republic of Korea; 8 Division of Hematology-Oncology, Department of Medicine, Samsung Medical Center, Sungkyunkwan University School of Medicine, Seoul 06351, Republic of Korea

**Keywords:** prognostic value, radiomics, ^18^F-FDG PET/CT, laryngeal cancer, hypopharyngeal cancer

## Abstract

Background: The prognostic value of conducting ^18^F-FDG PET/CT imaging has yielded different results in patients with laryngeal cancer and hypopharyngeal cancer, but these results are controversial, and there is a lack of dedicated studies on each type of cancer. This study aimed to evaluate whether combining radiomic analysis of pre- and post-treatment ^18^F-FDG PET/CT imaging features and clinical parameters has additional prognostic value in patients with laryngeal cancer and hypopharyngeal cancer. Methods: From 2008 to 2016, data on patients diagnosed with cancer of the larynx and hypopharynx were retrospectively collected. The patients underwent pre- and post-treatment ^18^F-FDG PET/CT imaging. The values of ΔPre-Post PET were measured from the texture features. Least absolute shrinkage and selection operator (LASSO) Cox regression was used to select the most predictive features to formulate a Rad-score for both progression-free survival (PFS) and overall survival (OS). Kaplan–Meier curve analysis and Cox regression were employed to assess PFS and OS. Then, the concordance index (C-index) and calibration plot were used to evaluate the performance of the radiomics nomogram. Results: Study data were collected for a total of 91 patients. The mean follow-up period was 71.5 mo. (8.4–147.3). The Rad-score was formulated based on the texture parameters and was significantly associated with both PFS (*p* = 0.024) and OS (*p* = 0.009). When predicting PFS, only the Rad-score demonstrated a significant association (HR 2.1509, 95% CI [1.100–4.207], *p* = 0.025). On the other hand, age (HR 1.116, 95% CI [1.041–1.197], *p* = 0.002) and Rad-score (HR 33.885, 95% CI [2.891–397.175], *p* = 0.005) exhibited associations with OS. The Rad-score value showed good discrimination when it was combined with clinical parameters in both PFS (C-index 0.802–0.889) and OS (C-index 0.860–0.958). The calibration plots also showed a good agreement between the observed and predicted survival probabilities. Conclusions: Combining clinical parameters with radiomics analysis of pre- and post-treatment ^18^F-FDG PET/CT parameters in patients with laryngeal cancer and hypopharyngeal cancer might have additional prognostic value.

## 1. Introduction

Laryngeal cancer is the second most common cancer of the head and neck regions, with an estimated 12,380 new cases and 3820 related deaths in the United States in 2023. Hypopharyngeal cancer is less common but its prevalence is still significant, with an annual incidence of approximately 3000 cases in the United States [1]. Patients with laryngeal cancer have a 5-year survival rate of 59%. Hypopharyngeal cancer presents late and patients have a 5-year survival rate of 25% to 40% [2]. Squamous cell carcinoma is predominant in most laryngeal and hypopharyngeal tumors. It is more common in the male population and is closely linked to heavy smoking. Optimal treatment to improve patients’ survival and preserve function involves a combination of surgical treatment, chemotherapy, and radiation therapy [3]. 

Analysis of the prognostic value of ^18^F-FDG PET/CT imaging showed various results in patients with head and neck cancer. Among the results reported, pre-treatment nodal SUVmax was an independent prognostic factor for recurrence [4]. SUVmax has its disadvantages, as it represents the most intense FDG uptake and may not reflect the total uptake of the whole tumor mass. Researchers have explored the prognostic relevance of various metabolic parameters derived from ^18^F-FDG PET in head and neck cancer. However, the outcomes have shown inconsistency, particularly in the literature on laryngeal cancer and hypopharyngeal cancers, which is relatively scarce [5]. 

Radiomics is an image analysis approach which aims to extract texture features of large data volumes from clinical medical images using a variety of mathematical techniques. The texture features include quantitative data of the histogram, intensity, and shape [6]. Numerous studies have explored the utilization of texture features extracted from ^18^F-FDG PET/CT scans to predict various aspects of disease progression [7], local failure [8,9], and overall survival [10,11,12]. However, there is a notable gap in research specifically focused on laryngeal or hypopharyngeal cancer in the literature on head and neck cancer. Moreover, while many studies predominantly rely on baseline PET/CT scans, a growing body of oncological research suggests that incorporating serial ^18^F-FDG PET/CT scans provides a more comprehensive reflection of a patient’s clinical outcomes [13,14,15]. To the best of our knowledge, there is no firm study on the prognostic value of serial ^18^F-FDG PET/CT in patients with laryngeal cancer and hypopharyngeal cancer. 

This study was undertaken to (1) assess whether radiomics provides supplementary prognostic value beyond established clinical parameters and (2) investigate the application of partial volume correction in the field of radiomics research pertaining to laryngeal cancer and hypopharyngeal cancer.

The purpose of this study was to investigate the prognostic significance of integrating radiomic analysis of both pre- and post-treatment ^1^F-FDG PET/CT imaging with clinical parameters in patients diagnosed with laryngeal cancer and hypopharyngeal cancer.

## 2. Materials and Methods

### 2.1. Patient Enrollment

We retrospectively reviewed patients who were diagnosed with laryngeal cancer and hypopharyngeal cancer at the Samsung Medical Center from 2008 to 2016. All of the patients received radiation therapy with or without concurrent chemotherapy. The inclusion criteria for our study were as follows: patients who underwent ^18^F-FDG PET/CT before and after receiving treatment; patients who had histopathologically proven squamous cell carcinoma. Exclusion criteria were as follows: patients who had no delineation of FDG uptake or no physiological uptake; patients who had a follow-up of less than 6 months.

We collected the following clinical data: age, sex, smoking history, T stage, N stage, history of induction chemotherapy, history of concurrent chemotherapy, and cancer type. The primary endpoint was PFS and the secondary endpoint was OS. 

This study was conducted according to the guidelines of the Declaration of Helsinki and was approved by the Institutional Review Board of the Samsung Medical Center Institutional Review Board (IRB No. 2020-09-185). The requirement for informed patient consent was waived.

### 2.2. Acquisition of ^18^F-FDG PET/CT Imaging 

All of the patients fasted for at least 6 h and were confirmed to have a blood glucose level of <200 mg/dL before scanning. Whole-body PET and CT images were performed approximately 60 min after injecting 5.0 MBq/kg ^18^F-FDG on a Discovery LS or a Discovery STE PET/CT scanner (GE Healthcare, Milwaukee, WI, USA). We conducted continuous spiral CT using either an 8-slice helical CT scan (140 keV, 40–120 mA; Discovery LS) or a 16-slice helical CT scan (140 keV, 30–170 mA; Discovery STE). Emission scans covered the area from the vertex to the proximal thigh, lasting for 4 min per frame in 2D mode for Discovery LS, and 2.5 min per frame in 3D mode for Discovery STE.

To generate PET images, we employed the CT scan data for attenuation correction through the ordered subset expectation maximization (OSEM) algorithm. This process involved 28 subsets and 2 iterations, utilizing a matrix size of 128 × 128 and a voxel size of 4.3 × 4.3 × 3.9 mm for Discovery LS. For Discovery STE, the PET images were reconstructed using the OSEM algorithm with 20 subsets and 2 iterations, along with a matrix size of 128 × 128 and a voxel size of 3.9 × 3.9 × 3.3 mm.

### 2.3. Feature Extraction Protocols

For quantitative analysis, the volumes of interest (VOIs) corresponding to the primary tumor were delineated using a semi-automated approach in the gradient-based algorithm known as PET Edge, integrated within MIM version 7.1.7 (MIM Software Inc., Cleveland, OH, USA). These delineated VOIs were also saved in DICOM-RT structure format. Subsequently, these VOIs were imported into the Chang-Gung Image Texture Analysis toolbox (available at http://code.google.com/p/cgita, accessed on 18 July 2012). This import and analysis procedure was performed by utilizing MATLAB software (version 2012a; MathWorks, Inc., Natick, MA, USA), which facilitated the extraction of texture features from the PET images. All radiomic parameters were based on the Imaging Biomarker Standardization Initiative (IBSI) guideline. Among them, we selected local texture features while considering the partial volume effect [16,17]. Overall, 40 tumoral heterogeneity indices (7 co-occurrence matrices, 6 normalized co-occurrence matrices, 13 SUV statistics, 2 texture spectra, 4 texture feature codings, and 8 texture feature coding co-occurrence matrices) were analyzed from CGITA software v1.0 (Supplementary Table S1).

### 2.4. Feature Selection and Rad-Score

We evaluated the LASSO regression algorithm to identify the most useful prognostic features among the extracted PET-based texture features based on the association between texture parameters and patient survival. To calculate the ΔPre-Post PET values, we subtracted the pre-PET measurement from the post-PET measurement and subsequently divided the result by the pre-PET measurement. 

We also employed n-fold cross-validation in our analysis to mitigate the risk of overfitting. Because LASSO shrinks the effect of unimportant features and can set their effects to zero while removing redundancy among the features, texture features with non-zero coefficients can be obtained. 

Subsequently, Rad-scores for predicting PFS and OS were calculated using the following formula:

$$Radscore=\overset{n}{\underset{i=1}{\sum }}\begin{array}{c} Coefficient\;of\;feature\;\left(i\right)\\ \times \;value\;of\;feature\;(i) \end{array}$$

where the coefficient of the radiomics feature (i) was the coefficient determined in the regression model. Patients were classified into high-risk and low-risk groups by an optimal cut-off value using X-tile analyses [18].

### 2.5. Model Construction and Evaluation

A flow chart of the study design is presented in Supplementary Figure S1. The patients were randomly divided into a training set and a validation set in a 2:1 ratio. All feature analyses were performed on the training set and subsequently validated on the validation set.

The discrimination ability of the combination of the clinical model and radiomics model was determined, and the predictive performance was compared by using the C-index. Kaplan–Meier curve analysis of PFS and OS based on the optimal cut-off value was performed to classify patients into high-risk and low-risk groups.

The calibration performance was assessed to predict PFS and OS using a calibration plot, which described the agreement between the predicted and observed survival probability. The nomogram transformed the corresponding model into a simple visual graph so that the results of the model were more distinct and of high clinical value.

### 2.6. Statistical Analysis

The clinical characteristics’ distribution within the patient cohort was illustrated by presenting the mean ± standard deviation. Variations in variables between the groups were assessed through an independent sample *t*-test and a chi-square test, selected based on the variables’ type and distribution.

For the statistical analysis, R software (version 4.2.2) was employed, along with specific R packages catering to distinct analytical tasks. The “glmnet” package facilitated the execution of LASSO Cox regression analysis, while differences among patients in the high-risk and low-risk groups were compared using the log-rank test. Cox regression analysis was used for both univariable analyses. The calibration curve and nomogram were established using Orange software (version 3.34.0). A two-sided *p*-value < 0.05 was considered to be statistically significant for all of the statistical analyses.

## 3. Results

### 3.1. Patient Characteristics 

Data for a total of 99 patients who were diagnosed with laryngeal cancer and hypopharyngeal cancer were collected. They all underwent ^18^F-FDG PET/CT before and after treatment. After excluding patients who were diagnosed with squamous cell carcinoma in situ (*n* = 5), withdrew from this study due to technical issues (*n* = 1), and had a follow-up period of less than 6 months (*n* = 2), a total of 91 patients were included in the final analyses. The baseline characteristics of the patients are presented in Table 1. 

No significant differences were detected between the training and validation sets regarding the following factors: age, sex, smoking history, cancer site, implementation of concurrent chemoradiotherapy or induction chemotherapy, T stage, N stage, mean progression-free survival, and mean overall survival. 

The mean follow-up period was 71.5 months (8.4–147.3). Of the 91 patients, 31 progressed and 26 died. The 3-year and 5-year progression-free survival estimate was 71% and 68%, respectively. The 3-year and 5-year overall survival estimate was 87% and 79%, respectively. 

### 3.2. Derivation of Rad-Score Formula from Radiomics Features

The VOIs of tumors from pre- and post-treatment FDG PET/CT images were measured and their texture features were evaluated. The LASSO Cox regression model was applied to choose the most significant features for survival analysis. 

Three and five potential predictors with non-zero coefficients were selected for predicting PFS and OS, respectively (*Normalized_Cooccurance_Second_angular_moment*, *Cooccurance_Correlation*, and *SUV_statistics_SUV_Variance* in the PFS model; *Cooccurance_Contrast*, *SUV_statistics_SUV_Variance*, *SUV_statistics_SUV_Kurtosis*, *SUV_statistics_SUV_bias_corrected_Skewness*, and *Texture_Feature_Coding_Cooccurance_Second_angular_moment* in the OS model). The minimum lambdas of 0.084 and 0.054 were assessed for predicting PFS and OS, respectively. The Rad-score formulas for the PFS and OS models were calculated as shown in the Supplementary Figure S2. According to the optimum cut-off value, all of the patients were classified into the high-risk group or the low-risk group in PFS and OS, respectively. 

**Figure 1:**
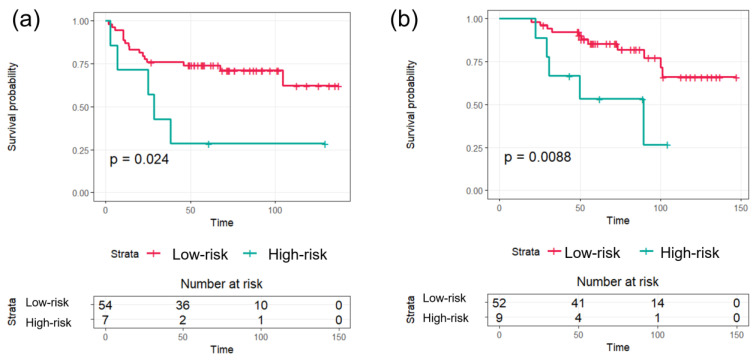
Kaplan–Meier analyses of the radiomics scores according to risk groups with progression-free survival (**a**) and overall survival (**b**) in training group.

Rad-score was significantly associated with both PFS and OS. In predicting PFS, the 3- and 5-year PFS of the low-risk group (76% and 74%, respectively) were higher than those of the high-risk group (43% and 29%, respectively, *p* = 0.024, Figure 1a). In predicting OS, the 3- and 5-year OS of the low-risk group (92% and 85%, respectively) were significantly higher than those of the high-risk group (67% and 53%, respectively, *p* = 0.009, Figure 1b).

The association between clinical characteristics and a risk-stratified group of radiomics according to the PFS and OS prediction models was investigated (Supplementary Tables S2 and S3). In the PFS prediction model, there were no differences in the clinical parameters between the low-risk and high-risk groups. In the PFS prediction model, only the number of those in the progression group (*p* = 0.030) were significantly different between the low-risk and high-risk groups. In the OS prediction model, there were significant differences in T stage (*p* = 0.042) and the number of deaths (*p* = 0.032) between the low-risk and high-risk groups. Otherwise, there was no significant difference between low-risk and high-risk groups in age, sex, smoking history, cancer site, concurrent chemoradiotherapy, induction chemotherapy, and N stage.

### 3.3. Estimating Prognostic Factors for Clinical Characteristics and Rad-Score

Survival analysis results using Cox’s method for estimating prognostic factors are presented in Table 2 and Table 3. In predicting PFS, only the Rad-score was associated with PFS (HR 2.1509, 95% CI [1.100–4.207], *p* = 0.025). On the other hand, in predicting OS, age (HR 1.116, 95% CI [1.041–1.197], *p* = 0.002) and Rad-score (HR 33.885, 95% CI [2.891–397.175], *p* = 0.005) were associated with OS.

The predictive performance, as measured by the C-index, is presented in Table 4 and Table 5. These performance metrics were applied to both the clinical model and the combined clinical and radiomics model. In the analysis of the PFS model, the C-index of the clinical model is shown for both the training (C-index 0.758, 95% CI [0.629 to 0.860]) and validation (C-index 0.895, 95% CI [0.724 to 0.977]) sets. In the analysis of the OS model, the C-index of the same model is presented for both the training (C-index 0.791, 95% CI [0.665 to 0.886]) and validation (C-index 0.916, 95% CI [0.752 to 0.986]) sets. Upon integration of the radiomics model, there was an increase in the C-index observed in both the training (C-index 0.860, 95% CI [0.745 to 0.937]) and validation (C-index 0.958, 95% CI [0.811 to 0.998]) sets for the overall survival (OS) model. Additionally, the C-index showed improvement in the training set (C-index 0.802, 95% CI [0.678 to 0.894]) for the progression-free survival (PFS) model. However, in the case of the validation set for the PFS model, there was a slight decrease in the C-index (C-index 0.889, 95% CI [0.717 to 0.975]).

### 3.4. Calibration Curve and Nomogram

**Figure 2:**
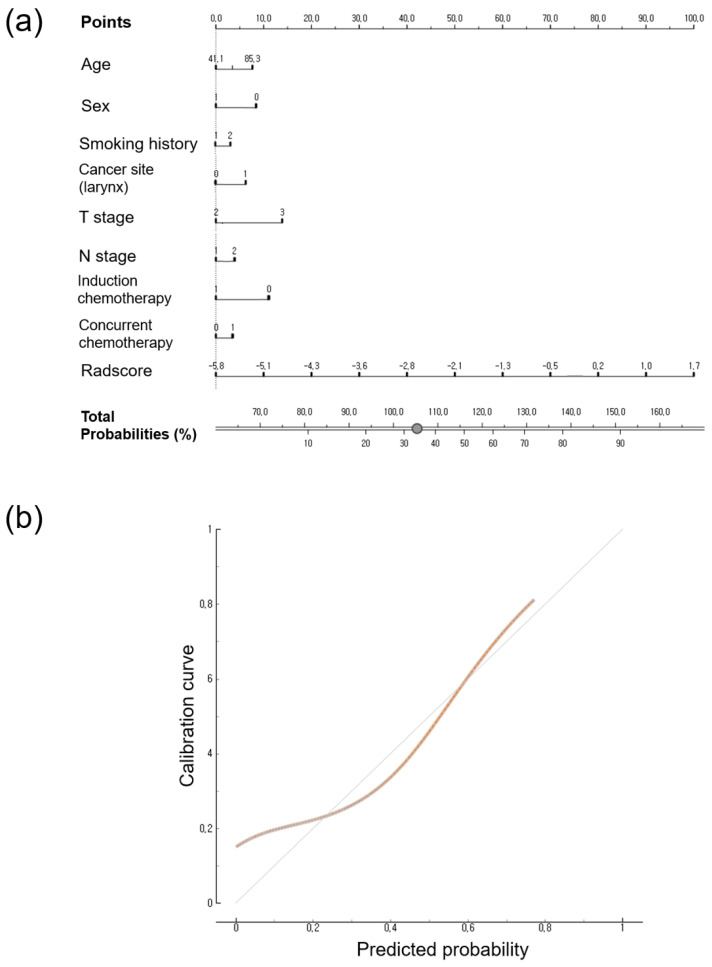
(**a**) Nomogram to predict the risk of 3-year disease progression-free survival. (**b**) Calibration curve depicted in terms of agreement between predicted and observed 3-year outcomes. The 45-degree diagonal line represents ideal calibration, where the predicted probabilities perfectly match the observed outcomes. The brown line represents the actual prediction of 3-year outcomes.

**Figure 3:**
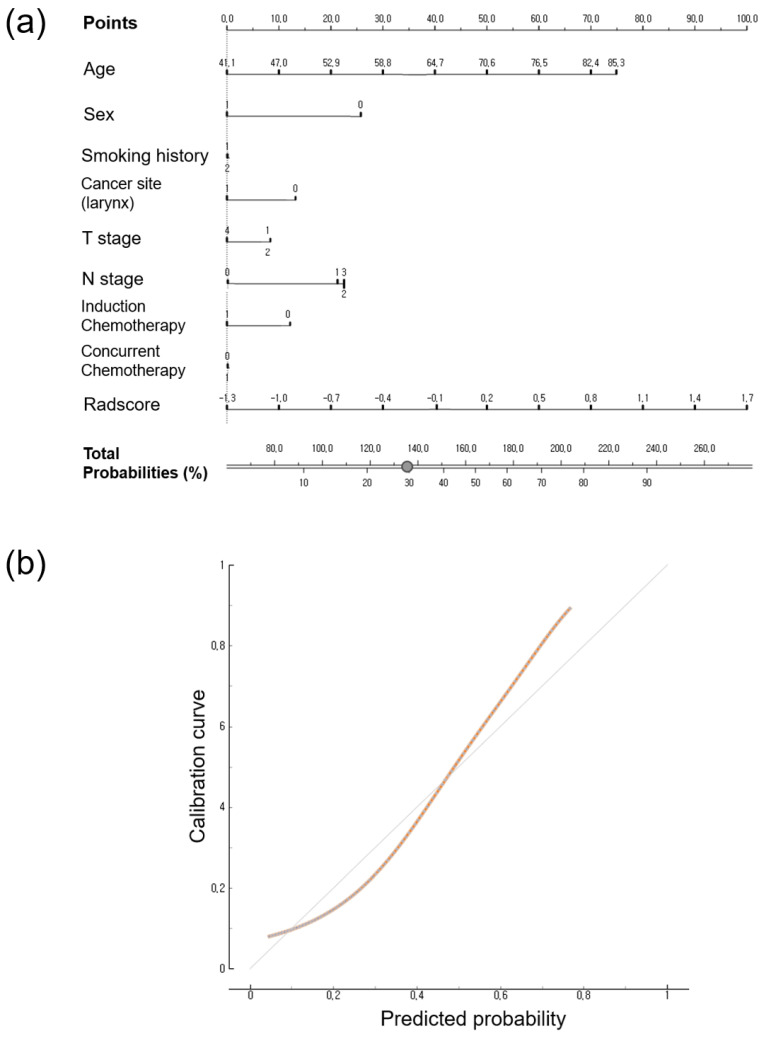
(**a**) Nomogram to predict the risk of 3-year overall survival. (**b**) Calibration curve depicted according to agreement between predicted and observed 3-year outcomes. The 45-degree diagonal line represents ideal calibration, where the predicted probabilities perfectly match the observed outcomes. The brown line represents the actual prediction of 3-year outcomes.

The nomogram and calibration of the Rad-score and clinical data for predicting PFS and OS are shown in Figure 2 and Figure 3. The total scores were used to predict the probability of PFS and OS by integrating the individual scores for age, sex, T stage, N stage, induction chemotherapy, concurrent chemotherapy, smoking history, cancer type, and Rad-score. The calibration curves at 3 years showed good agreement between predicted probability and observed values in PFS and OS, respectively. The cumulative probabilities for predicting a poorer progression-free survival were aggregated based on the factors presented in Figure 2, namely, increasing age, male gender, smoking history, presence of hypopharyngeal cancer, higher T and N stages, lack of induction chemotherapy, receiving concurrent chemotherapy, and a higher Rad-score. Similarly, an increased likelihood of predicting poorer overall survival was observed in relation to advancing age, male gender, higher T and N stages, absence of induction chemotherapy, and a higher Rad-score (Figure 3).

## 4. Discussion

This study aimed to investigate whether texture analysis using pre- and post-treatment PET/CT scans can provide additional prognostic value for patients who underwent definitive radiotherapy/concurrent radiotherapy for laryngeal cancer and hypopharyngeal cancer. Our results showed that Rad-scores derived from radiomics features were significantly associated with both PFS and OS. Rad-score was the only prognostic factor for the analysis of PFS, whereas age was added to the prognostic factors for OS. Our results also indicate that the combination of Rad-scores and clinical parameters may offer predictive performance for both PFS and OS. Furthermore, we presented calibration plots that demonstrated good results, along with a nomogram that enhanced the clinical value and distinctiveness of the model’s results.

An efficient evaluation strategy for patients with laryngeal cancer is needed due to the complicated regional anatomy, significant anatomical structure of the adjacent regions, critical structural changes related to treatment response, and various intratumoral heterogeneity [19]. In this study, a calculation method was adopted that excluded variables sensitive to partial volume correction. Regional variables (intensity variability and size zone variability) were excluded, as they were more sensitive to partial volume correction than local parameters (co-occurrence, SUV statistics) [16]. First-order and second-order texture features were selected for enrollment. First-order texture features are the frequency distribution of tumors of one-voxel intensity such as conventional PET parameters. The second-order texture features are calculated based on the local association of delineated tumors with intensities of two voxels [17]. Implementing partial volume correction in clinical settings has shown potential for enhancing prognostication in individuals diagnosed with head and neck cancer [20]. 

There are several studies on the use of radiomic analysis to predict survival in patients with head and neck cancer [7,10,11]. Zhong et al. reported favorable results from combining PET and CT parameters of extracted texture features for predicting disease progression after treatment [7]. They suggested metabolic tumor volume (MTV) and SUVmin as PET parameters. Liu et al. included post-treatment PET/CT for analysis, although their interpretation was limited to negative/positive only. The results showed that a combination of clinicopathologic characteristics and radiomics features had better prediction power than clinicopathologic characteristics alone. 

The findings of this investigation are similar to those reported by Martens et al. [21]. Specifically, they underscore the significance of first-order parameters, such as SUVmean, in the prediction of outcomes related to recurrence, distant metastasis, and overall survival. Furthermore, Martens et al. extended their analysis to incorporate HPV status in the prediction of time-to-event outcomes. However, it is noteworthy that one study introduced voxel-alignment features, focusing on low-intensity long-run emphasis, to predict local control through the utilization of a LASSO regression method [8]. It is important to recognize that this particular study had inclusion criteria limited to patients with clinical stages 3 or 4, in contrast to our study, which included patients with lower stage disease. This disparity suggests that radiomic information in advanced cancer cases may exhibit a stronger association with higher order texture features, particularly concerning local invasion pathways and heterogeneous pathophysiology. 

Numerous studies have explored the utility of delta-radiomic analyses to assess treatment response across different cancer types and imaging modalities, yielding a range of outcomes [22,23,24,25]. Nevertheless, the interpretation of their findings remains challenging due to the inherent opacity of radiomics models, often referred to as a “black-box” approach. Another factor contributing to this complexity is the inherent pathophysiology of tumors, because radiomics features, while informative as prognostic factors, may undergo changes that do not necessarily align with the tumor’s actual biological characteristics. We also propose that our study protocol was carefully designed to account for the partial volume effect before applying LASSO Cox regression for feature selection. It is important to note that the results could potentially differ if the sequence of these steps was reversed.

Investigating within the realm of head and neck cancer prognostic factors, an emerging consideration is the ABO blood type [26,27,28,29]. One possible theory involves the potential disruption of or imbalance in the enzymatic activity of ABO glycosyltransferases. The particular function of these enzymes lies in facilitating intercellular adhesion and signaling across cellular membranes. Shifting these surface molecules might contribute to the progression of malignancy [26,30]. Nonetheless, it is essential to point out that the findings may not consistently align across various studies, and further research is often necessary to establish conclusive and universally applicable prognostic markers.

This study has several limitations. First, data were collected retrospectively. Thus, selection bias might affect the study results. The result of pre-treatment PET/CT affecting the regimen of treatment cannot be excluded in this setting. Information bias, like incomplete or inaccurate records, might skew the data and impact the reliability of results. Second, two different PET/CT scanners were used in this study, which might have caused the inconsistency in texture analysis. However, the gradient-based PET edge delineation method might represent accurate and reproducible information, as it is less affected by camera resolution, reconstruction method, and filtering than manual or other automatic delineation techniques [31,32]. Third, the feature extraction software utilized in the present study is not fully compliant with Imaging Biomarker Standardization Initiative (IBSI) guidelines. Nevertheless, the terminology and equations for texture features, as well as the calculation procedures, are in line with the recommendations outlined by the IBSI [33]. The software has already undergone external validation in studies predicting treatment response in breast cancer [34] and has been utilized in various cancer types for diagnosis [33,35], treatment response [13], and prognosis [36,37] in numerous studies. 

## 5. Conclusions

This study aimed to evaluate whether combining radiomic analysis of pre- and post-treatment (radiotherapy/concurrent radiotherapy) ^18^F-FDG PET/CT imaging features and clinical parameters has additional prognostic value in patients with laryngeal cancer and hypopharyngeal cancer. Rad-scores derived from radiomics scores showed prognostic power in both PFS and OS. Rad-score was significantly associated with PFS and OS. In combination with clinical factors, Rad-scores can potentially show the discriminative power of a risk prediction model in both PFS and OS.

## Figures and Tables

**Table 1:** Demographics and clinicopathologic characteristics of patients.

Characteristics	All Patients (*n* = 91)	Training Set (*n* = 61)	Validation Set (*n* = 30)	*p* Value
Mean age, y	62.50 ± 10.00	61.34 ± 9.55	65.00 ± 10.70	0.103
Sex (M:F)				0.798
Male	84	56	28	
Female	7	5	2	
Smoking history				0.349
Ex/current smoker	77	53	24	
Never smoked	11	6	5	
N/A	3			
Cancer site				0.924
Larynx	57	38	19	
Hypopharynx	34	23	11	
Concurrent chemoradiotherapy				0.201
Yes	57	41	16	
No	34	20	14	
Induction chemotherapy				0.984
Yes	6	4	2	
No	85	57	28	
T stage				0.317
T1	20	11	9	
T2	41	28	13	
T3	18	14	4	
T4	12	8	4	
N stage				0.575
N0	47	30	17	
N1	17	16	1	
N2	26	15	11	
N3	1	0	1	
Mean ^a^ PFS, mo	59.30 ± 40.32	61.15 ± 38.85	55.53 ± 43.59	0.535
Mean ^b^ OS, mo	72.10 ± 35.51	74.22 ± 33.27	67.78 ± 39.93	0.420

^a^ PFS: progression-free survival; ^b^ OS: overall survival.

**Table 2:** Univariable Cox regression analyses for predicting progression-free survival in the training cohort.

Variables	Hazard Ratio (95%CI)		*p*-Value
Age	1.0245	(0.974–1.077)	0.347
Sex (F/M)	0.6419	(0.086–4.790)	0.666
Smoking history (ex/current smoker/never smoked)	1.8968	(0.551–6.534)	0.310
T2	0.5173	(0.145–1.847)	0.310
T3	1.6504	(0.495–5.503)	0.415
T4	0.9511	(0.213–4.256)	0.948
N1	0.6646	(0.208–2.123)	0.490
N2	1.5761	(0.597–4.162)	0.359
Induction chemotherapy (yes/no)	0.6609	(0.089–4.932)	0.686
Concurrent chemotherapy (yes/no)	1.5537	(0.568–4.250)	0.391
Cancer type (hypopharyngeal cancer/laryngeal cancer)	1.4726	(0.623–3.481)	0.378
Rad-score	2.1509	(1.100–4.207)	0.025

**Table 3:** Univariable Cox regression analyses for predicting overall survival in the training cohort.

Variables	Hazard Ratio (95%CI)		*p*-Value
Age	1.1162	(1.041–1.197)	0.002
Sex (F/M)	1.36 × 10^−8^	(0–Inf)	0.998
Smoking history (ex/current smoker/never smoked)	0.9612	(0.123–7.504)	0.970
T2	0.7856	(0.186–3.316)	0.743
T3	2.3993	(0.589–9.779)	0.222
T4	1.1261	(0.187–6.790)	0.897
N1	1.1485	(0.335–3.938)	0.826
N2	1.6463	(0.520–5.208)	0.396
Induction chemotherapy (yes/no)	1.0813	(0.143–8.198)	0.940
Concurrent chemotherapy (yes/no)	1.2448	(0.429–3.610)	0.687
Cancer type (hypopharyngeal cancer/laryngeal cancer)	0.7829	(0.271–2.260)	0.651
Rad-score	33.885	(2.891–397.175)	0.005

**Table 4:** Evaluating the predictive performance for the analysis of progression-free survival.

Model	C-Index (95% CI)	
	Training Set	Validation Set
Clinical model	0.758 (0.629 to 0.860)	0.895 (0.724 to 0.977)
Clinical + Radiomics model	0.802 (0.678 to 0.894)	0.889 (0.717 to 0.975)

**Table 5:** Evaluating the predictive performance for the analysis of overall survival.

Model	C-Index (95% CI)	
	Training Set	Validation Set
Clinical model	0.791 (0.665 to 0.886)	0.916 (0.752 to 0.986)
Clinical + Radiomics model	0.860 (0.745 to 0.937)	0.958 (0.811 to 0.998)

## Data Availability

The datasets generated during and/or analyzed during the current study are available from the corresponding author on reasonable request.

## Supplementary Materials

The following supporting information can be downloaded at: https://www.mdpi.com/article/10.3390/jpm14010071/s1, Table S1: Radiomics features extracted from CGITA; Figure S1: Flow chart of the study design; Figure S2: Rad-score formula; Table S2: The clinical data of risk-stratified groups according to PFS model in training set; Table S3: The clinical data of risk-stratified groups according to OS model in training set.
