# Correction: Choi et al. Prognostic Value of Radiomic Analysis Using Pre- and Post-Treatment ^18^F-FDG-PET/CT in Patients with Laryngeal Cancer and Hypopharyngeal Cancer. *J. Pers. Med.* 2024, *14*, 71

**DOI:** 10.3390/jpm15020074

**Published:** 2025-02-19

**Authors:** Joon Ho Choi, Joon Young Choi, Sang-Keun Woo, Ji Eun Moon, Chae Hong Lim, Soo Bin Park, Seongho Seo, Yong Chan Ahn, Myung-Ju Ahn, Seung Hwan Moon, Jung Mi Park

**Affiliations:** 1 Department of Nuclear Medicine, Soonchunhyang University Bucheon Hospital, Bucheon 14584, Republic of Korea; 2 Department of Nuclear Medicine, Samsung Medical Center, Sungkyunkwan University School of Medicine, Seoul 06351, Republic of Korea; 3 Division of Applied RI, Korea Institutes of Radiological and Medical Sciences, Seoul 01812, Republic of Korea; 4 Department of Biostatistics, Soonchunhyang University Bucheon Hospital, Bucheon 14584, Republic of Korea; 5 Department of Nuclear Medicine, Soonchunhyang University Seoul Hospital, Seoul 04401, Republic of Korea; 6 Department of Electronic Engineering, Pai Chai University, Daejeon 35345, Republic of Korea; 7 Department of Radiation Oncology, Samsung Medical Center, Sungkyunkwan University School of Medicine, Seoul 06351, Republic of Korea; 8 Division of Hematology-Oncology, Department of Medicine, Samsung Medical Center, Sungkyunkwan University School of Medicine, Seoul 06351, Republic of Korea

In the original publication [1], there was a biographical error where an incorrect citation was included as Reference [35]. With this correction, the originally published citation for Reference [35] has been removed and replaced with the following: 

Ji, Y.; Qiu, Q.; Fu, J.; Cui, K.; Chen, X.; Xing, L.; Sun, X. Stage-Specific PET Radiomic Prediction Model for the Histological Subtype Classification of Non-Small-Cell Lung Cancer. *Cancer Manag. Res.* **2021**, *13*, 307–317.

This correction was approved by the Academic Editor. The original publication has also been updated.
