# Supplementary material for: Prognostic Value of Radiomic Analysis Using Pre- and Post-Treatment 18F-FDG-PET/CT in Patients with Laryngeal Cancer and Hypopharyngeal Cancer
Source: J Pers Med. 2024 Jan 5;14(1):71. doi: 10.3390/jpm14010071 (PMC10817325; doi:10.3390/jpm14010071)
Supplement: Supplementary file 1 [file jpm-14-00071-s001.zip › jpm-2761517-supplementary.pdf]

Supplementary Table S1. Radiomics features extracted from [a]CGITA.

| Parent matrix of CGITA | Feature measure of CGITA |
|---|---|
| Co-occurrence | Second angular moment |
| Co-occurrence | Contrast |
| Co-occurrence | Entropy |
| Co-occurrence | Homogeneity |
| Co-occurrence | Dissimilarity |
| Co-occurrence | Inverse difference moment |
| Co-occurrence | Correlation |
| Normalized Co-occurrence | Second angular moment |
| Normalized Co-occurrence | Contrast |
| Normalized Co-occurrence | Entropy |
| Normalized Co-occurrence | Homogeneity |
| Normalized Co-occurrence | Dissimilarity |
| Normalized Co-occurrence | Inverse difference moment |
| SUV statistics | Minimum SUV |
| SUV statistics | Maximum SUV |
| SUV statistics | Mean SUV |
| SUV statistics | SUV Variance |
| SUV statistics | SUV SD |
| SUV statistics | SUV Skewness |
| SUV statistics | SUV Kurtosis |

| | |
|---|---|
| SUV statistics | SUV bias-corrected Skewness |
| SUV statistics | SUV bias-corrected Kurtosis |
| SUV statistics | TLG |
| SUV statistics | Tumor volume |
| SUV statistics | Entropy |
| SUV statistics | SULpeak |
| Texture Spectrum | Max spectrum |
| Texture Spectrum | Black–white symmetry |
| Texture Feature Coding | Coarseness |
| Texture Feature Coding | Homogeneity |
| Texture Feature Coding | Mean convergence |
| Texture Feature Coding | Variance |
| Texture Feature Coding Co-occurrence | Second angular moment |
| Texture Feature Coding Co-occurrence | Contrast |
| Texture Feature Coding Co-occurrence | Entropy |
| Texture Feature Coding Co-occurrence | Homogeneity |
| Texture Feature Coding Co-occurrence | Intensity |
| Texture Feature Coding Co-occurrence | Inverse difference moment |
| Texture Feature Coding Co-occurrence | Code Entropy |
| Texture Feature Coding Co-occurrence | Code Similarity |

[a]CGITA : Chang-Gung Image Texture Analysis

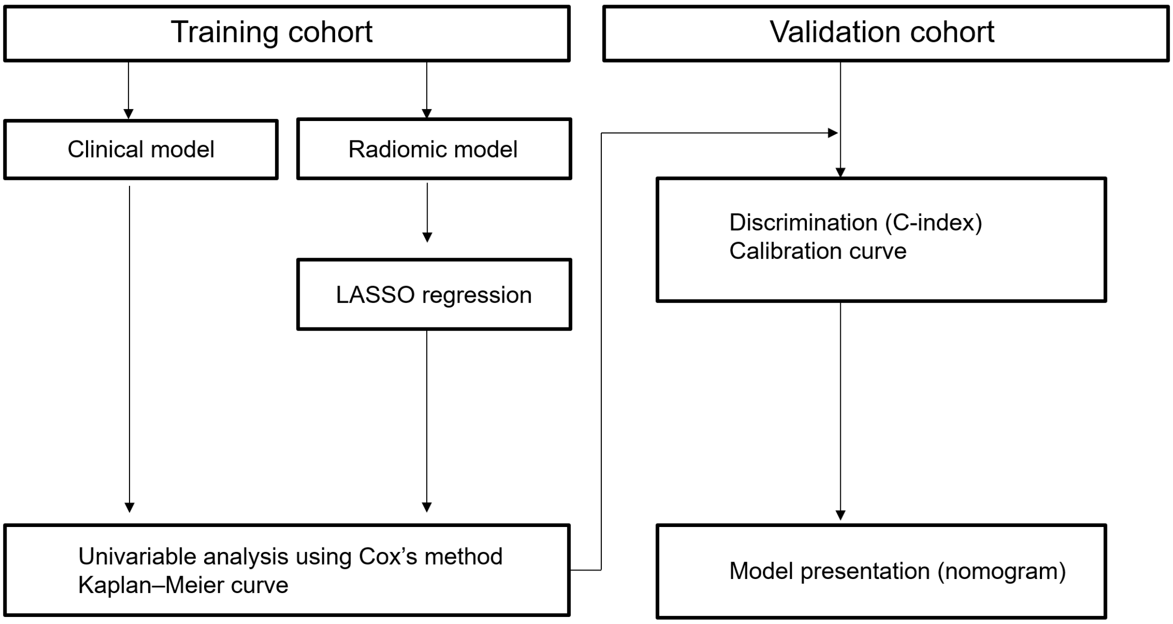


Supplementary Figure S1. Flow chart of the study design.

Predicting PFS:

Rad-score

= Normalized_Cooccurance_Second_angular_moment * 0.0015226635

+ Cooccurance_Correlation * 0.0091515752

+ SUV_statistics_SUV_Variance * 0.0031195177

Predicting OS:

Rad-score

= Cooccurance_Contrast *-0.0041757495

+ SUV_statistics_SUV_Variance * 0.0013239587

+ SUV_statistics_SUV_Kurtosis *-0.0015628803

+ SUV_statistics_SUV_bias_corrected_Skewness * 0.0006349189

+ Texture_Feature_Coding_Cooccurance_Second_angular_moment * 0.0005972383

Supplementary Figure S2. Rad-score formula.

Supplementary Table S2. The clinical data of risk-stratified groups according to [a]PFS model in training set.

| Characteristic | Low-risk group (n=54) | High-risk group (n = 7) | p |
|---|---|---|---|
| Mean age, y | 61.46 ± 9.24 | 60.41 ± 12.50 | 0.785 |
| Sex | | | 0.405 |
| male | 49 (91%) | 7 (100%) | |
| female | 5 (9%) | 0 (0%) | |
| Smoking history | | | 0.347 |
| Ex/current smoker | 46 (85%) | 7 (100%) | |
| Never smoked | 6 (12%) | 0 (0%) | |
| N/A | 2 (3%) | 0 (0%) | |
| Cancer site | | | 0.178 |
| Larynx | 32 (59%) | 6 (86%) | |
| Hypopharynx | 22 (41%) | 1 (14%) | |
| Concurrent chemoradiotherapy | | | 0.148 |
| Yes | 38 (70%) | 3 (43%) | |
| No | 16 (30%) | 4 (57%) | |
| Induction chemotherapy | | | 0.460 |
| Yes | 4 (7%) | 0 (0%) | |
| No | 50 (93%) | 7 (100%) | |
| T stage | | | 0.607 |

| | | | |
|---|---|---|---|
| T1 | 8 (15%) | 3 (43%) | |
| T2 | 26 (48%) | 2 (29%) | |
| T3 | 14 (26%) | 0 (0%) | |
| T4 | 6 (11%) | 2 (29%) | |
| N stage | | | 0.536 |
| N0 | 25 (46%) | 5 (71%) | |
| N1 | 16 (30%) | 0 (0%) | |
| N2 | 13 (24%) | 2 (29%) | |
| N3 | 0 (0%) | 0 (0%) | |
| No. of progression | | | 0.030 |
| Yes | 16 (30%) | 5 (71%) | |
| No | 38 (70%) | 2 (29%) | |

[a]PFS: progression-free survival.

Supplementary Table S3. The clinical data of risk-stratified groups according to [b]OS model in training set.

| Characteristic | Low-risk group (n=52) | High-risk group (n = 9) | P |
|---|---|---|---|
| Mean age, y | 60.78 ± 9.46 | 64.58 ± 9.95 | 0.274 |
| Sex | | | 0.732 |
| male | 48 (92%) | 8 (89%) | |
| female | 4 (8%) | 1 (11%) | |
| Smoking history | | | 0.198 |
| Ex/current smoker | 46 (88%) | 7 (78%) | |
| Never smoked | 4 (8) | 2 (12%) | |
| N/A | 2 (4%) | 0 (0%) | |
| Cancer site | | | 0.654 |
| Larynx | 33 (63%) | 5 (56%) | |
| Hypopharynx | 19 (37%) | 4 (44%) | |
| Concurrent chemoradiotherapy | | | 0.468 |
| Yes | 34 (65%) | 7 (78%) | |
| No | 18 (35%) | 2 (22%) | |
| Induction chemotherapy | | | 0.553 |
| Yes | 3 (6%) | 1 (11%) | |
| No | 49 (94%) | 8 (89%) | |
| T stage | | | 0.042 |

| | | | |
|---|---|---|---|
| T1 | 10 (19%) | 1 (12%) | |
| T2 | 26 (50%) | 2 (22%) | |
| T3 | 11 (21%) | 3 (33%) | |
| T4 | 5 (10%) | 3 (33%) | |
| N stage | | | 0.336 |
| N0 | 26 (50%) | 4 (44%) | |
| N1 | 15 (29%) | 1 (12%) | |
| N2 | 11 (21%) | 4 (44%) | |
| N3 | 0 (0%) | 0 (0%) | |
| No. of death | | | 0.032 |
| Yes | 11 (21%) | 5 (56%) | |
| No | 41 (79%) | 4 (44%) | |

[b]OS: overall survival.
